# Historical evidence of glyphosate exposure from a US agricultural cohort

**DOI:** 10.1186/s12940-019-0474-6

**Published:** 2019-05-07

**Authors:** Melissa J. Perry, Daniele Mandrioli, Fiorella Belpoggi, Fabiana Manservisi, Simona Panzacchi, Courtney Irwin

**Affiliations:** 1 Department of Environmental and Occupational Health, Milken Institute School of Public Health, The George Washington University, 950 New Hampshire Ave, Washington, DC, 20052 USA; 2 Cesare Maltoni Cancer Research Center (CMCRC), Ramazzini Institute (RI), Via Saliceto, 3, 40010 Bentivoglio, Bologna Italy

**Keywords:** Glyphosate, AMPA, Biomonitoring, Occupational epidemiology, Environmental epidemiology, Toxicology, Agricultural health, Farmers, Urinalysis, Pesticides

## Abstract

In response to the recent review by Gillezeau et al., *The evidence of human exposure to glyphosate: A review*, *Environmental Health 1/19/19*, here we report additional glyphosate biomonitoring data from a repository of urine samples collected from United States farmers in 1997–98. To determine if glyphosate exposure could be identified historically, we examined urine samples from a biorepository of specimens collected from US dairy farmers between 1997 and 98. We compared samples from farmers who self-reported glyphosate application in the 8 h prior to sample collection to samples from farm applicators who did not report using glyphosate. Of 18 applicator samples tested, 39% showed detectable levels of glyphosate (mean concentration 4.04 μg/kg; range:1.3–12) compared to 0% detections among 17 non glyphosate applicator samples (*p*-value < 0.01). One of the applicator samples that tested positive for glyphosate also tested positive for AMPA. Concentrations of glyphosate were consistent with levels reported in the prior occupational biomonitoring studies reviewed by Gillezeau et al.

Accurately detecting both glyphosate and AMPA in this small sample of Wisconsin farmers demonstrates a) glyphosate exposures among farmers were occurring 20 years ago, which was prior to the widespread planting of genetically engineered glyphosate tolerant crops first approved in 1996; and b) liquid chromatography tandem mass spectrometry (LC-MS/MS) can be used for sensitive characterization in cryopreserved urine samples. These data offer an important historical benchmark to which urinary levels from current and future biomonitoring studies can be compared.

## Main text

Gillezeau et al.’s [1] review of glyphosate biomonitoring published in this journal earlier this year demonstrated that few glyphosate biomonitoring studies have been conducted since 2007. Because of glyphosate’s widespread use and frequent presence in the environment worldwide, it is important to understand routes and cumulative levels of exposure and potential human-health effects.

Between 1971 and 2014, the US applied nearly 1.6 billion kilograms of glyphosate, 66% of which had been applied in the previous decade [2]. Glyphosate-based herbicides (GBHs) are approved for over 100 different agricultural and non-agricultural uses including weed control along roads, canals, railroads, powerlines; in and around commercial and industrial facilities; and in a myriad of public spaces and around homes. GBHs have been detected in air, water, food, and companion and farm animal feedstuffs [3] and it can remain in food for over a year even after washing, cooking, and freezing practices [4].

In 1996, Roundup Ready, a glyphosate-tolerant seed type, was approved for farm use in the US and has since been integrated into the global genetically modified (GM) crop industry. Today, the US grows a variety of glyphosate-resistant plants including soybeans, maize, cotton, alfalfa, and sugarbeets. In 2012, approximately 94% of all soybeans (30 million hectares) planted in the US were Roundup Ready [5].

There are few glyphosate biomonitoring studies to date largely because the analytic methods for detecting glyphosate and its metabolite aminomethylphosphonic acid (AMPA) have not been well established. This is likely due to previous assumptions that glyphosate was non toxic based on prior genotoxicity testing, could only be applied to crops pre emmergence, and did not pose an exposure risk via groundwater runoff or food residue [6]. More accurate and sensitive methods using mass spectrometry (MS) have advanced more recently, which can improve accurate expsoure assessment and evaluation of health effects. For example, a recently published study involving 71 women in central Indiana found that over 90% of the women studied had detectable glyphosate levels in their urine; a finding that was significantly associated with a shorter pregnancy [7].

To determine if glyphosate exposure could be identified historically, we examined urine samples collected from dairy farmers between 1997 and 98 who self-reported glyphosate application.

## Methods

Urine samples were collected following consent from farmers working on family farms in Wisconsin between 1997 and 1998, and stored at − 80 C. Recruitment and screening procedures have been discussed in detail elsewhere [8]. Briefly, urine samples were collected by farmers 8 h following first of the season pesticide application on cropland. Because the main study focused on restricted use pesticides only and because urinalysis measures were not readily available at the time of the original study, glyphosate was not analyzed in urine and no additional information beyond self-reported use was collected for glyphosate.

In 2018, we identified 18 samples from farmers who had reported applying glyphosate, and randomly selected 18 additional samples from the remaining archive of over 200 stored urines from the original study. Samples were analyzed by Neotron Laboratories to determine whether glyphosate or its metabolite, AMPA could be detected. Neotron used the US Food and Drug Administration’s liquid chromatography tandem mass spectrometry (LC-MS/MS) method [9] to detect and quantify glyphosate and AMPA residues. The limit of detection (LOD) for glyphosate was 0.4 μg/kg urine (0.4 ppb) and AMPA was 1 μg/kg urine (1 ppb).

Pearson Chi-square tests were used to compare detections among applicators and non applicators, and to determine if there was an association between glyphosate detection and farmer age, amount of land farmed, owned, or rented, gross annual farm income, hours of application of non GBH pesticides or number of acres to which non GBH pesticides were applied.

## Results

One non-applicator sample could not be analyzed, resulting in 18 applicator and 17 non-applicator samples for urinalysis. Seven of the 18 applicator and none of the 17 non applicator samples had glyphosate detections above the limit of detection (Χ^2^ = 8.3; *p*-value < 0.01).

Table 1 compares concentration levels found in this study to the 8 occupational studies reviewed by Gillezeau et al. [1]. The mean level of glyphosate detected among Wisconsin dairy farmers was 4.04 μg/kg (4.04 ppb) across the seven positive samples (range 1.3–12.0 μg/kg). One individual, who had the highest glyphosate concentration (12.0 μg/kg), also had AMPA detected above the LOD (4.1 μg/kg). No other AMPA detections were observed.

**Table 1:** Comparison of occupational biomonitoring studies for glyphosate in urine, 1991–2018

	Time of Collection	Method	Number of samples tested	Creatinine	Glyphosate Average Concentration	LOD	% above LOD	AMPA	LOD	% above LOD
Present Study (2019) United States	After application	LC-MS/MS	35	uncorrected	4.04 ppb	0.1 ppb	39	4.1 ppb	0.1 ppb	6
Jauhainen et al. (1991) Finland	After application	GC	5	uncorrected	<LOD	100 ppb	0	NR	NR	NR
Acquavella et al. (2004) United Staes	Spot samples	HPLC	48	uncorrected	3.2 ppb	1 ppb	60	NR	NR	NR
Curwin et al. (2007) United States	Spot samples	FCMIA	24	corrected	1.6 ppb	0.9 ppb	23	NR	NR	NR
Mesnage et al. (2012) France	After application	LC-MS	1	uncorrected	9.5 ppb	1 ppb	20	NR	NR	NR
	Two days later		1		2 ppb	1 ppb	20	NR	NR	NR
Jayasumana et al. (2015) Sri Lanka	Spot samples	ELISA	10	corrected	73.5 ppb	0.5 ppb		NR	NR	NR
Connolly et al. (2017) Ireland	Before application	LC MS/MS	18	corrected	0.71 ppb	0.5 ppb	35	NR	NR	NR
	After application		18		1.35 ppb	0.5 ppb	55	NR	NR	NR
Rendon von Osten et al. (2017) Mexico	Spot samples	ELISA	81	uncorrected	0.22–0.47 ppb^a^	0.05 ppb	NR	NR	NR	NR
Connolly et al. (2018) Ireland	Before use	LC MS/MS	20	corrected	1.08 ppb	0.5 ppb	62	NR	NR	NR
	After use		20		1.72 ppb	0.5 ppb	82	NR	NR	NR
	Morning void		20		1.32 ppb	0.5 ppb	62	NR	NR	NR
	Peak sample		20		2.53 ppb	0.5 ppb	93	NR	NR	NR

*AMPA* aminomethylphosphonic acid, *ELISA* enzyme-linked immunosorbent assay, *FCMIA* Fluorescence covalent microbead immunoassay, *HPLC* High-performance liquid chromatography, *LC* Liquid chromatography, *LOD* limit of detection, *MS* mass spectrometry, *MS/ MS* tandem mass spectrometry, *NR* not reported, *PPB* parts per billion

^a^study reported a concentration range, not average

There was no association between glyphosate detection and farmer age, amount of land farmed, owned, or rented, gross annual farm income, hours of application of non GBH pesticides or number of acres to which non GBH pesticides were applied.

## Discussion

Of the 35 farmer applicators whose urine was tested for glyphosate and AMPA, none of the non applicators had positive detections, whereas 39% of the applicators had positive detections. The sample with the highest concentration of 12.0 μg/kg also tested positive for AMPA. Both findings support the sensitivity of LC MS/MS to detect the actual analyte and its metabolite.

The occupational biomonitoring studies conducted to date and summarized in Table 1 demonstrate a) most have been conducted with small samples (range 1–76 participants; average 22); b) LODs vary widely, from 100 to 0.5 ppb; c) percent detections >LOD also vary widely (range 20–100%); c) there has a been a marked decrease in LODs over time; and d) none of the prior studies measured AMPA. Also of note is that creatinine adjustment has been used inconsistently, the importance of which for accurate glyphosate characterization in human urine is unclear [10].

Newly published reports are demonstrating previously unrecognized health impacts of glyphosate exposure, including a meta analysis of epidemiologic studies showing a link between glyphosate based herbicide expsoure and increased risk of Non-Hodgkin Lymphoma [11] and a long-term low dose toxicologic assessment that showed glyphosate and Roundup had developmental and endocrinological impacts in sprague dawley rats [12].

Precise measurements of glyphosate in human urine and serum is critical for informing human-equivalent doses in experimental models, particularly at low doses. Importantly, recent evidence from in vivo investigations suggests glyphosate bioaccumulates in rats, which requires further exposure assessment and biomarker investigation in humans [13]. Glyphosate is never used alone and is combined with adjuvants to increase plant penetration which can be more toxic than glyphosate alone [14]. To fully characterize health effects, adjuvants need to be identified and biomonitoring assessments need to be able to detect them in conjunction with glyphosate and AMPA.

While the effects of long-term specimen storage on glyphosate molecular integrity has not been well studied (see [15] for a 1998 report using nuclear magnetic resonance spectroscopy for detecting glyphosate proteins using flash freezing), the impacts of different freezing methods with and without cryoprotectants on glyphosate recovery need to be further assessed experimentally, and with other historical urine specimen repositories.

Detecting both glyphosate and AMPA in this small sample of Wisconsin farmers demonstrates LC-MS/MS can be used to detect concentrations in urine samples undergoing long-term cryopreservation (samples remained in -80c without cryoprotectant since first collection), and that glyphosate exposures among US farmers were occurring 20 years ago.

## Abbreviations

AMPA: Aminomethylphosphonic acid
CDC: Centers for Disease Control and Prevention
EFSA: European Food Safety Authority
GBH: Glyphosate Based Herbicide
GM: Genetically modified
IARC: International Agency for Research on Cancer
LC-MS/MS: Liquid chromatography mass spectrometry
LOD: Limit of Detection
MS: Mass spectrometry
NHANES: National Health and Nutrition Examination Survey

## References

[1] Gillezeau C, Van Gerwen M, Shaffer RM, Rana I, Zhang L, Sheppard L, Taioli E. The evidence of human exposure to glyphosate: a review. Environ Health. 2019;18(2). 10.1186/s12940-018-0435-5 PMC6322310 30612564

[2] Benbrook CM (2016). Trends in glyphosate herbicide use in the United States and globally. Environ Sci Eur.

[3] Van Bruggen AHC, He MM, Shin K, Mai V, Jeong KC, Finckh MR, Morris JG. Environmental and health effects of the herbicide glyphosate. Sci Total Environ. 2018;616-617:255. 10.1016/j.scitotenv.2017.10.309 29117584

[4] Krüger M, Schledorn P, Schrödl W, Hoppe H-W, Lutz W, Shehata AA (2014). Detection of glyphosate residues in animals and humans. J Environ Anal Tox.

[5] United States Department of Agriculture. National Agricultural Statistics Service. Agricultural chemical usage—field crops and potatoes 2014. https://usda.library.cornell.edu/concern/publications/2n49t1699?locale=en. Accessed 27 Jan 2019.

[6] Myers JP, Antoniou MN, Blumberg B, Carroll L, Colborn T, Everett LG, Hansen M, Landrigan PJ, Lanphear BP, Mesnage R, Vandenberg L, Vom Saal FS, Welshons WV, Benbrook CM (2016). Concerns over use of glyphosate-based herbicides and risks associated with exposures: a consensus statement. Environ Health.

[7] Parvez S, Gerona RR, Proctor C, Friesen M, Ashby JL, Reiter JL, Lui Z, Winchester PD (2018). Glyphosate exposure in pregnancy and shortened gestational length: a prospective Indiana birth cohort study. Environ Health.

[8] Perry MJ, Christiani DC, Mathew J, Degenhardt D, Tortorelli J, Strauss J, Sonzogni WC (2000). Urinalysis of atrazine exposure among farm pesticide applicators. Toxicol Ind Health.

[9] United States Food and Drug Administration (2002). Pesticide Analytical Manual Vol. II -Method I: Gas chromatographic determination of residues of Fosetyl-Al and phosphorous acid in pineapples. Pesticide Reg. Sec.

[10] Connolly Alison, Jones Kate, Basinas Ioannis, Galea Karen S., Kenny Laura, McGowan Padraic, Coggins Marie A. (2019). Exploring the half-life of glyphosate in human urine samples. International Journal of Hygiene and Environmental Health.

[11] Zhang L, Rana L, Shaffer RM, Taioli E, Sheppard L. Exposure to glyphosate-based herbicides and risk for non-Hodgkin lymphoma: a meta-analysis and supporting evidence. Mutat Res online 10 Feb, 2019. 10.1016/j.mrrev.2019.02.001 PMC6706269 31342895

[12] Manservisi F, Lesseur C, Panzacchi S, Mandrioli D, Falcioni L, Luciano B, Manservigi M, Spinaci M, Galeati G, Mantovani A, Lorenzetti S, Miglio R, Andrade AM, Kristensen DM, Perry MJ, Swan SH, Chen J, Belpoggi F (2019). The Ramazzini institute 13-week pilot study glyphosate-based herbicides administered at human-equivalent dose to Sprague Dawley rats: effects on development and endocrine system. Environ Health.

[13] Panzacchi S, Mandrioli D, Manservisi F, Bua L, Falcioni L, Spinaci M, Galeati G, Dinelli G, Miglio R, Mantovani A, Lorenzetti S, Hu J, Chen J, Perry MJ, Landrigan PJ, Belpoggi F (2018). The Ramazzini institute 13-week study on glyphosate-based herbicides at human equivalent dose in Sprague Dawley rates: study design and first in-life endpoints evaluation. Environ Health.

[14] Valle AL, Mello FCC, Alves-Balvedi RP, Rodrigues LP, Goulart LR (2019). Glyphosate detection: methods, needs and challenges. Environ Chem Lett.

[15] Jakeman D, Mitchell DJ, Shuttleworth WA, Evans JNS (1998). Effects of sample preparation conditions on biomolecular solid-state NMR lineshapes. J Biomolecular NMR.

